# Divide, Conquer, and Sense: CD8^+^CD28^−^ T Cells in Perspective

**DOI:** 10.3389/fimmu.2016.00665

**Published:** 2017-01-03

**Authors:** Fernando A. Arosa, André J. Esgalhado, Carolina A. Padrão, Elsa M. Cardoso

**Affiliations:** ^1^Health Sciences Research Centre (CICS-UBI), Universidade da Beira Interior, Covilhã, Portugal; ^2^Faculty of Health Sciences (FCS-UBI), Universidade da Beira Interior, Covilhã, Portugal

**Keywords:** effector memory CD8^+^ T cells, NK-like T cells, natural killer receptors, innate receptors, open MHC-I conformers, IL-15, IFN-γ, tissue repair

## Abstract

Understanding the rationale for the generation of a pool of highly differentiated effector memory CD8^+^ T cells displaying a weakened capacity to scrutinize for peptides complexed with major histocompatibility class I molecules *via* their T cell receptor, lacking the “signal 2” CD28 receptor, and yet expressing a highly diverse array of innate receptors, from natural killer receptors, interleukin receptors, and damage-associated molecular pattern receptors, among others, is one of the most challenging issues in contemporary human immunology. The prevalence of these differentiated CD8^+^ T cells, also known as CD8^+^CD28^−^, CD8^+^KIR^+^, NK-like CD8^+^ T cells, or innate CD8^+^ T cells, in non-lymphoid organs and tissues, in peripheral blood of healthy elderly, namely centenarians, but also in stressful and chronic inflammatory conditions suggests that they are not merely end-of-the-line dysfunctional cells. These experienced CD8^+^ T cells are highly diverse and capable of sensing a variety of TCR-independent signals, which enables them to respond and fine-tune tissue homeostasis.

## Preface

Thanks to their T cell antigen receptor (TCR), thymus-derived CD8^+^ T cells have the unique ability to scrutinize any cell of our body displaying at the cell surface peptides bound to major histocompatibility class I (MHC-I) molecules and respond by means of cell activation and proliferation whenever the MHC-I molecule looks different than usual. In this regard, the quote “Divide and Conquer” (from the Latin saying *Divide et Impera*, credited to Julius Caesar) is the name of an algorithm that solves a problem by breaking it sequentially into two or more sub-problems until these become simple enough to be solved (1). Paraphrasing the quote and the algorithm, it can be anticipated that the tendency of a thymus-derived CD8^+^ T cell to divide and generate a progeny of cells is meant to solve a problem, keep body homeostasis, by making conquerors that travel to distant injured/inflamed tissues (effector CD8^+^ T cells), some of which may mutiny and become a problem (inflammatory CD8^+^ T cells) and have to be restrained by peacekeepers (suppressor/regulator CD8^+^ T cells). At the end of the process, a mixture of the different subsets survives as sensors of any further change that may occur within the environment they visited and conquered (memory CD8^+^ T cells). Human CD8^+^ T cell differentiation is a complex process enfolded in contrasting views on the functional role of the memory CD8^+^ T cells under normal and diseased conditions. Hereby, we present a perspective on the function of these CD8^+^ T cells that focus on the relationship with their internal environment.

## CD8^+^ T Cell Differentiation: One-Way Ticket to Pleiotropy

Before leaving the thymus to enter the circulation, CD8^+^ T cells survive two critical events that determine their fate in the periphery. First, they learn to *trans*-interact *via* their TCR clonotypic receptor with composites of a MHC-I heavy chain, a light chain (β_2_m), and a short peptide (2). These antigen-presenting MHC-I structures are also designated “closed conformers” to distinguish them from the “open conformers” that are constituted only by the MHC-I heavy chain after dissociation from the light chain and/or the peptide and that can exist at the cell surface in an ordered non-denatured form (3). Open conformers can interact in *trans* and *cis* with a variety of receptors, namely members of the natural killer receptor (NKR) family, with important functional implications, as discussed below. The recognition of closed MHC-I conformers gives naïve CD8^+^ T cells the capacity to survive in the periphery and eventually recognize and be activated by closed MHC-I conformers presenting an excess of unusual antigens (4). After activation, naïve CD8^+^ T cells enter differentiation programs that result in the generation of effector CD8^+^ T cells displaying different bioactivities (5). After the excess of antigen is neutralized and removed, homeostatic mechanisms are turned on to cease the effector function while keeping a small pool that remains in circulation as memory CD8^+^ T cells (6). Second, CD8^+^ T cells are genetically programed to express an array of receptors during the differentiation process, which allows them to receive activation and survival signals from receptors and ligands other than MHC class I closed conformers (3, 7–10).

**Figure 1:**
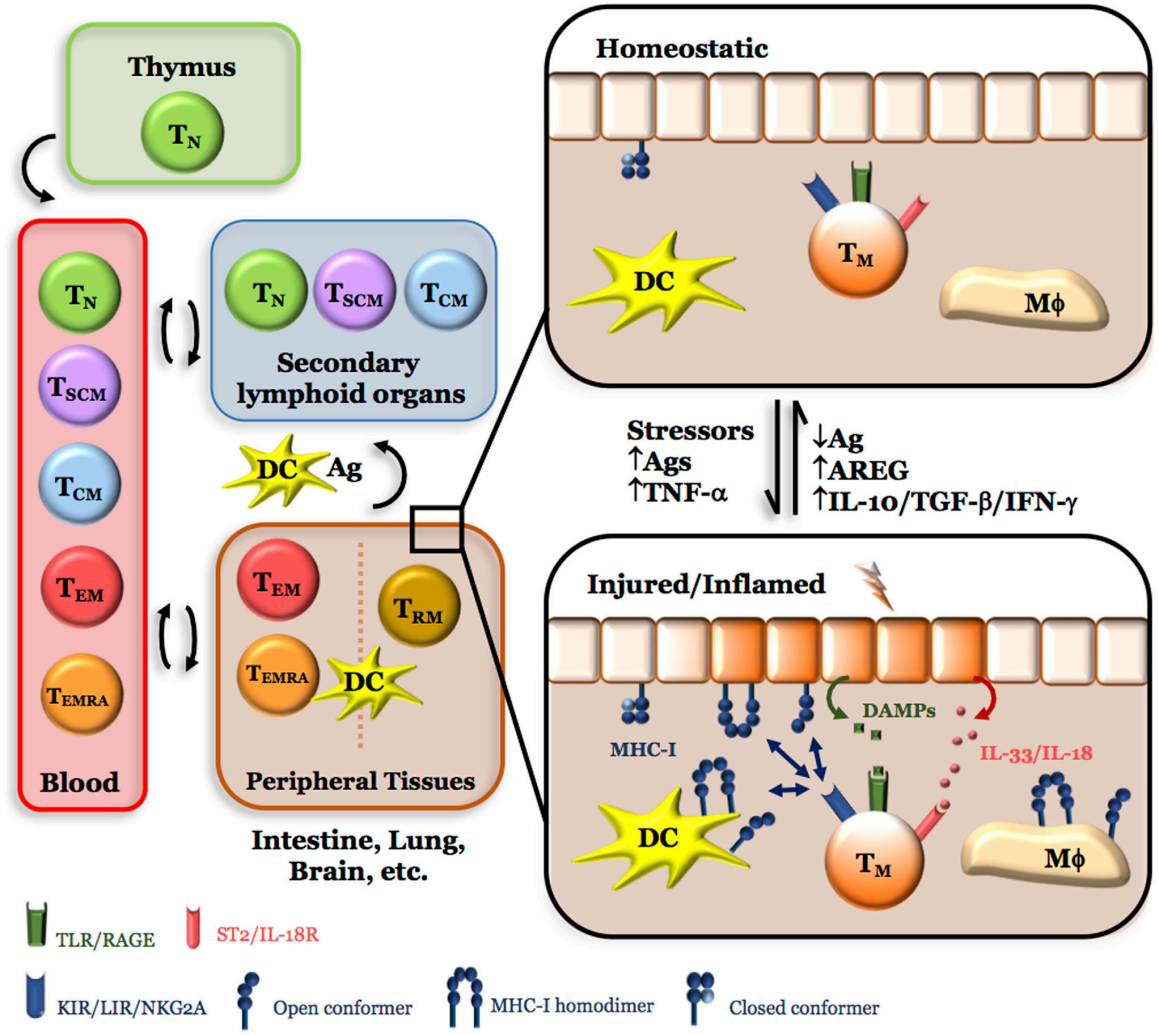
**Simplified model for the role of NK-like CD8^+^ Tem cells in tissue integrity**. Of the five major circulating CD8^+^ T cell pools, naive (Tn), stem-cell memory (Tscm), and central memory (Tcm) preferentially migrate to secondary lymphoid organs, where they can be activated by processed antigens presented by closed major histocompatibility class I (MHC-I) conformers expressed by dendritic cells (DC) recently arrived from peripheral tissues and differentiate into effector memory (Tem) and effector memory CD45RA^+^ (Temra). On the other hand, CD8^+^ Tem and Temra have preferential, but not exclusive, access to peripheral tissues under homeostatic (healthy) conditions where they can stay as CD8^+^ Trm. Under tissue stress and/or injury, a sudden increase in antigens (Ags) and/or inflammatory cytokines (TNF-α) results in the release of endogenous products [damage-associated molecular patterns (DAMP), IL-33, ATP, etc.] and expression of open MHC-I conformers by immune and non-immune cells. While tissue DCs could migrate to secondary lymphoid organs and induce more cycles of CD8^+^ T cell activation and differentiation, Tem, Temra, and Trm (denoted as Tm for simplicity) could directly sense these changes *in loco*; thanks to the expression of killer Ig-like receptor, leukocyte Ig-like receptor, NKG2A, DAMP receptors, IL-18/IL-33 receptors, purinergic receptors, and others. Thus, the presence of CD8^+^ Tm cells in peripheral tissues allows a faster response to harmful situations by secreting cytokines (IFN-γ, IL-10, TGF-β) and factors [amphiregulin (AREG)] that activate pathways leading to tissue repair and regeneration, and, therefore, to the homeostatic (healthy) state. Any imbalance in this equilibrium (e.g., overt tissue injury and necrosis, hypoxia, excess of antigen, high CD4/CD8 T cell ratios, low numbers or absence of CD8^+^ Tem, Temra, Trm cells, etc.) will result in a failure to resolve inflammation and chronic inflammation will ensue.

As a result of the huge effort done during the last decades and based on the expression of CCR7, CD27, CD28, CD45RA, and others, we have now a close picture of the main differentiation stages of human CD8^+^ T cells (Figure 1). Thus, the recirculating peripheral CD8^+^ T cell compartment is a mixture of lymphocytes distributed among five major pools: naïve (Tn), stem-cell memory (Tscm), central memory (Tcm), effector memory (Tem), and effector memory CD45RA^+^ (Temra) (11–13). An additional pool of non-recirculating tissue-resident memory cells (Trm) has also been described (14). Despite certain phenotypic and functional overlap among these CD8^+^ T cell pools, this classification has been most useful to describe the level of differentiation that the CD8^+^ T cell compartment has endured under different inflammatory settings, such as autoimmunity, cancer, and acute and chronic viral responses (15–17). Yet, perhaps the most significant achievement has been the identification of genes differently expressed by these pools, allowing to envision novel roles for CD8^+^ T cells (7, 18–20).

The CD8^+^ Tn pool comprises polyclonal T cells recently emigrated from the thymus that express CD28, CCR7, and CD62L, the two latter allowing them to recirculate between blood and secondary lymphoid organs (21). The CD8^+^ Tem and CD8^+^ Temra pools (for easiness, both termed as Tem thereafter) are highly differentiated CD8^+^ T cells that differ in the expression of the tyrosine phosphatase isoform CD45RA. They were formerly described as lymphocytes lacking CD28, responding poorly to TCR-stimulation, displaying redirected cytotoxicity, containing oligoclonal T cells, and being able to migrate to non-lymphoid organs and tissues (21–23). CD8^+^ T cells with the Tem phenotype were reported to express receptors thought to be solely expressed by NK cells, including CD56, CD94/NKG2A, killer Ig-like receptors (KIR), and leukocyte Ig-like receptors (LIR), among others (24–26). CD8^+^ T cells with the Tem phenotype also express NKp46 (27), a member of the natural cytotoxicity receptor, akin to several inhibitory receptors, such as CTLA-4, PD1, TIM3, and LAG3 (28). Due to these distinguishing features, they have also been designated CD8^+^CD28^–^, CD8^+^KIR^+^, CD8^+^NKR^+^, NK-like CD8^+^ T cells, and more recently innate CD8^+^ T cells (29–32). The evidence gathered during recent years suggests that the human CD8^+^ Tem pool is very diverse and polyfunctional and contains cells endowed with suppressor, inflammatory, and cytotoxic features (25–35). Whether these polyfunctional CD8^+^ Tem cells reflect the existence of distinctive subsets or a pleiotropic CD8^+^ T cell population that displays its activities depending on the signals that receive in the different tissue environments, remains to be elucidated.

## On the Origin of NK-Like CD8^+^ T Cells: Aging, Viruses, Cytokines, and More

Following the initial description of CD8^+^CD28^−^ T cells in the late 1980s and early 1990s, high levels of these cells were described in peripheral blood of healthy elderly people, during viral infections (e.g., CMV, HIV, and EBV), cancer, and autoimmunity (8). Nowadays, alterations in CD8^+^CD28^−^ T cells have been reported in almost every chronic inflammatory disease. Studies performed on CMV-seropositive elderly showed that a sizable fraction of CD8^+^CD28^−^ T cells contains CMV-specific CD8^+^ T cells (17). The description in the elderly of an association between the accumulation of CD8^+^CD28^−^ T cells, a phenomenon called memory CD8^+^ T cell inflation (36), CMV seropositivity, a decrease in survival rate and faulty *in vivo* humoral and cellular responses to vaccination, brought about the view that CD8^+^ Tem cells were terminally differentiated dysfunctional cells that contributed to immunosenescence and susceptibility to develop chronic inflammatory diseases (35–40). Recent studies are revealing that CMV-specific CD8^+^ Tem are not dysfunctional cells. Rather, they are polyfunctional in terms of cytokine secretion and proliferation (41–44), capable of surviving for longer periods of time (45, 46), and are only functionally restricted by the set of inhibitory receptors they express (28, 41). On the other hand, longitudinal studies comparing IgG titers and DNA viral load with CMV-specific CD8^+^ T cell frequencies suggest that CMV serology may not be a reliable indicator to study associations between chronic CVM infection and CD8^+^ Tem cell expansions (47). Thus, the association between CD8^+^ T cell expansions, CMV seropositivity, immunosenescence, and predisposition to disease remains an open question (48).

Besides chronic activation by viral antigens, there is solid evidence that cytokines such as IL-15, TNF-α, and TGF-β, as well as several cell types, regulate CD8^+^ T cell homeostasis. IL-15 displays multiple bioactivities, namely induction and maintenance of CD8^+^ Tem cells *in vitro* and *in vivo* (49–54), suggesting that memory CD8^+^ T cell inflation may also result from encounters with cytokines, thus increasing virtual memory CD8^+^ T cells (55). IL-15 is also involved in liver homeostasis and regeneration after hepatectomy (56). Whether this bioactivity is linked to the pro-survival activities of hepatocytes on CD8^+^ T cells and the presence of large amounts of CD8^+^ Tem cells in the liver, remains to be elucidated (27, 57). On the other hand, reconstitution studies in mice have shown that accumulation of CD8^+^ Tem cells depends on the presence of IL-15 and CD4^+^ T cells (9). Finally, intestinal epithelial cells and 4-1BBL^+^ B cells have also been shown to drive expansion and accumulation of CD8^+^ T cells with the Tem phenotype *in vitro* (58, 59) and *in vivo* (60), respectively, expanding the universe of factors that drive NK-like CD8^+^ T cell generation.

While unnoticed, expansions of CD8^+^ T cells with a Tem phenotype were described in conditions where oxidative stress is high, including HFE hemochromatosis, heavy alcohol consumption, hemodialysis, β-thalassemia, and during acute exercise (61–65). Although the molecular cues underlying CD8^+^CD28^−^ Tem generation under stressful conditions are uncertain, PGE2, a byproduct of arachidonic acid catabolism produced under pro-oxidant and inflammatory conditions, induces expression of NKG2A and downregulates CD28 on CD8^+^ T cells, two features associated with the acquisition of the Tem phenotype (66–68). Oxidative stress has also been shown to regulate expression of Bach2, a transcription factor involved in the formation of CD8^+^ Tem cells through downregulation of genes associated with effector function, such as Blimp1 (69, 70). On the other hand, expression of the transcription factor HIF-1 by CD8^+^ T cells *in vitro* under low oxygen conditions, mimicking acute exercise, correlates with accumulation of CD8^+^ Tem cells (71), which is in agreement with the reported role of HFI-1 in modulating the balance between effector and memory CD8^+^ T cells in models of chronic activation (72). These data suggest that oxidative stress may play an important role in modulating the formation of CD8^+^ Tem cells.

Finally, the CD4/CD8 T cell ratio is a factor that appears to influence the extent of the CD8^+^CD28^−^ T cell expansions. Early studies in HFE hemochromatosis and heavy alcohol drinkers reported a positive correlation between the size of the CD8^+^CD28^−^ T cell expansions and the size of the CD8^+^ T cell pool, regardless of age (61, 62). Importantly, the regression curve had a much higher slope and correlation coefficient in the patients than in the control group, implying that under stressful/adverse conditions there is a hastened formation of CD8^+^CD28^−^ T cells (8, 61, 62). Similar results were observed when the percentage of CD8^+^CD56^+^ T cells, which is increased in the elderly, was analyzed (73), strongly suggesting that the expansions of CD8^+^CD28^−^ T cells are constrained by the size of the CD8^+^ T cell pool in relation to the CD4^+^, which are both under the control of major autosomal recessive genes (74). The importance of this influence is illustrated by two sets of studies. First, studies in infants with overt CD4^+^ T cell lymphopenia and reversed CD4/CD8 T cell ratios, due to deficiency in the tyrosine kinase p56lck, showed that the peripheral CD8^+^ T cell pool was made up almost entirely of CD8^+^ T cells with the Tem phenotype (75–77). Second, a recent cross-sectional study in elderly people, including centenarians, showed that the heterogeneity found in the CD8^+^ Tem pool could be explained by variations in the size of the CD8^+^ T cell compartment (78). Although it is difficult to discern what is cause and what is effect, we favor the view that the CD4/CD8 T cell ratio influences the extent of the CD8^+^CD28^−^ T cell expansions. In this context, it is worth mentioning that the expansions CD8^+^CD28^−^ T cells reported in HFE hemochromatosis patients were paralleled by a defective CD8-associated p56lck (61, 79). In view of these data and studies in mice showing that CD8-associated Lck is dispensable for maintenance of memory CD8^+^ T cells (80), it is tempting to speculate that in humans a deficient CD8-p56lck signaling and expansion of CD8^+^CD28^−^ T cells could be intertwined processes.

## On the Function of NK-Like CD8+ T Cells: There is Life Beyond Closed MHC-I Conformers

The accumulated evidence indicates that loss of CD28, shrinkage of the TCR repertoire, gain of a variety of NKR, and expression of tissue homing receptors are interdependent events that end up in the formation of polyfunctional human CD8^+^ Tem cells that migrate to peripheral tissues where a fraction stays as a pool of non-recirculating CD8^+^ Trm cells upon expression of CD69 and CD103 (14). A series of recent studies using tissue samples from otherwise healthy infant and adult organ donors have shown that CD8^+^ Tem cells are predominant within non-lymphoid tissues and organs, including the brain, and this prevalence increases from childhood to adultness (81–84). CD8^+^ Tem predominance also occurs in the healthy bone marrow, stomach, and gingiva (85–87). Interestingly, lower CD4/CD8 T cell ratios within these tissues are associated with a larger CD8^+^ Tem pool (81), pointing again to the importance of the molecular cues that regulate this setting. Although recirculating and non-recirculating CD8^+^ Tem present in non-lymphoid tissues confer local immune protection against infections (88–90), it is also true that CD8^+^ Tem cells adapt to the new environment and may participate in the resolution of inflammation followed by tissue regeneration and repair after injury through complex networks involving cross-talk with other tissue environmental cells (91–99).

The picture emerges where CD28 loss and expression KIR, LIR and other NKR allows CD8^+^ Tem cells to engage in a cross-talk with other cells in their environment (8, 100, 101). But how this acquired skill is conveyed in terms of control of tissue integrity and organ function? As already mentioned, cell surface MHC-I molecules can exist in equilibrium between closed and open conformers, a process that is regulated by endocytosis and phosphorylation of a conserved tyrosine residue in the cytoplasmic tail of MHC-I heavy chains, and that allows the open conformers to self-associate and form novel structures called class I homodimers (3, 102–104). In this context, a series of recent reports are unveiling the many lives of MHC class I molecules (105), by showing that besides interacting with closed conformers in a peptide-independent manner (106, 107), KIR and LIR also interact with open conformers and homodimers (108–114). These results are of upmost importance if we consider that open conformers are expressed and/or released by metabolically active and stressed immune and non-immune cells, including neurons (103, 115–119). Thus, expression of NKR by CD8^+^ Tem cells allows them to sense changes in the level of closed and open MHC-I conformers, i.e., in the stressful/inflammatory state of the environment. The impact that KIR/LIR/NKG2A engagement has on CD8^+^ T cell survival and cytokine secretion (29, 52, 120), is of the foremost importance for tissue homeostasis under normal or pathological conditions. Thus, IFN-γ and other cytokines released by CD8^+^ Tem upon NKR triggering could mediate resolution of inflammation and subsequent tissue healing by modulating growth and proliferation of epithelial and other tissue cells (121, 122). Importantly, by inducing upregulation of classical and non-classical MHC-I molecules on epithelial/endothelial cells, IFN-γ may further promote survival and proliferation signals upon MHC-I reverse signaling by their cognate NKR expressed by CD8^+^ Tem cells (123), thus harnessing the healing process.

Although expression of NKR allows CD8^+^ Tem cells located in peripheral tissues to sense changes in the closed ↔ open MHC-I equilibrium, this is likely not enough to cope with the fluctuations that occur within an ever changing internal environment (Figure 1). In this respect, there is evidence that CD8^+^ Tem cells also express receptors for damage-associated molecular patterns (DAMP), which are specialized in recognizing endogenous products released by cell stress, injury, or dead (124). DAMP receptors include toll-like receptors (TLR), advance glycosylation end products receptors (RAGE), receptors for IL-1 family members (e.g., IL-18 and IL-33), purinergic receptors (P2YR), and β2-adrenergic receptors (19, 125–129), to cite some. Although most of the studies on these receptors have focused on innate cells, there is growing evidence that DAMP receptor expression by CD8^+^ Tem cells could broaden their capacity to sense the disruption of tissue homeostasis and respond by secreting regulatory cytokines and healing factors. Thus, ligation of TLR on CD8^+^ Tem cells is known to augment IFN-γ in *in vitro* and *in vivo* settings (130, 131), which in barrier tissues such as the lung, where a large fraction of CD8^+^ T cells are Temra (81) may exacerbate tissue pathology (132). RAGE encompasses multiple ligands, including glycated proteins, nuclear high-mobility group box 1 (HMGB1), S100 proteins, and β-amyloid, among others, which transmit intracellular signals associated with tissue repair and regeneration (133, 134). RAGE^+^CD8^+^ T cells were described more than two decades ago and proposed to participate in the regulation of tissue homeostasis through secretion of IFN-γ (135). HMGB1 can also bind to TIM3, an inhibitory receptor expressed on CD8^+^ Temra cells, whose inhibitory function depends on the co-expression of CEACAM-1 (28, 136). Although expression of the IL-18 and IL-33 receptors by CD8^+^ T cells is known for some time (127), their importance in the regulation of tissue repair by T cells has only recently emerged (137–139). Thus, binding of IL-18 and IL-33 to regulatory T cells triggers the secretion of amphiregulin (AREG), a ligand for the EGF receptor involved in suppression of inflammation and tissue repair (140, 141). Although formal proof for the secretion of AREG by CD8^+^ Tem cells is lacking, there is evidence that CD8^+^ T cells express this tissue repair factor (141, 142). Finally, CD8^+^ Tem cells also express purinergic receptors (19), as well as the β2-adrenergic receptor (129). While the former can sense environmental nucleotides released under adverse conditions and induce suppressive signals on T cells, thus downplaying inflammation (128, 143), the latter allows the sympathetic nervous system to communicate under stressful conditions with CD8^+^ T cells (144, 145).

## Concluding Remarks

Although one important facet of CD8^+^ T cells has to do with tissue damage and injury resulting from coping with infections, this should not overshadow other facets of CD8^+^ T cells related with the maintenance of tissue integrity and homeostasis (Figure 1). Since the description of CD8^+^CD28^−^ T cells about 25 years ago, a huge amount of information has been obtained on the functional phenotype and localization of these lymphocytes. Their capacity to migrate and reside in peripheral tissues in parallel with the expression of receptors for unconventional MHC-I molecules and endogenous products released by injured, inflamed, and necrotic cells may endow these cells with the capacity to fine-tune tissue repair, regeneration, and homeostasis by a number of ways, namely by inducing epithelial, endothelial, and mesenchymal cells to grow and proliferate and inhibiting inflammatory responses. All these bioactivities will likely involve active crosstalk with environmental cells and complex loops between secreted cytokines.

## Author Contributions

FA and EC wrote the manuscript. EC drew the figure. AE and CP read and edited the manuscript.

## Conflict of Interest Statement

The authors declare that the research was conducted in the absence of any commercial or financial relationships that could be construed as a potential conflict of interest.
